# Gene expression and DNA methylation are extensively coordinated with MRI-based brain microstructural characteristics

**DOI:** 10.1007/s11682-018-9910-4

**Published:** 2018-06-22

**Authors:** Chris Gaiteri, Robert Dawe, Sara Mostafavi, Katherine D. Blizinsky, Shinya Tasaki, Vitalina Komashko, Lei Yu, Yanling Wang, Julie A. Schneider, Konstantinos Arfanakis, Philip L. De Jager, David A. Bennett

**Affiliations:** 1 Rush Alzheimer’s Disease Center, Rush University Medical Center, Chicago, IL USA; 2 Department of Diagnostic Radiology and Nuclear Medicine, Rush University Medical Center, Chicago, IL USA; 3 Department of Statistics, Department of Medical Genetics, University of British Columbia, Vancouver, BC Canada; 4 National Institutes of Health, National Human Genome Research Institute, Bethesda, MD USA; 5 Department of Biomedical Engineering, Illinois Institute of Technology, Chicago, IL USA; 6 Columbia University College of Physicians and Surgeons, New York, NY USA

**Keywords:** Alzheimer’s disease, brain networks, molecular networks, gene expression, MRI

## Abstract

Cognitive function relies on both molecular levels and cellular structures. However, systematic relationships between these two components of cognitive function, and their joint contribution to disease, are largely unknown. We utilize postmortem neuroimaging in tandem with gene expression and DNA methylation, from 222 deeply-phenotyped persons in a longitudinal aging cohort. Expression of hundreds of genes and methylation at thousands of loci are related to the microstructure of extensive regions of this same set of brains, as assessed by MRI. The genes linked to brain microstructure perform functions related to cell motility, transcriptional regulation and nuclear processes, and are selectively associated with Alzheimer’s phenotypes. Similar methodology can be applied to other diseases to identify their joint molecular and structural basis, or to infer molecular levels in the brain on the basis of neuroimaging for precision medicine applications.

**Electronic supplementary material:**

The online version of this article (10.1007/s11682-018-9910-4) contains supplementary material, which is available to authorized users.

## Introduction

Molecular activity and brain structure influence each other (West and Greenberg 2011), and both affect cognitive function (Bishop et al. 2010). Progress towards a systematic molecular basis for neuroimaging findings indicates that regional volumes are shaped by genetic factors (Hibar et al. 2015), and that spatial patterns of gene expression correspond to cell type distributions (Krienen et al. 2016), structural (Fulcher and Fornito 2016) and functional connectivity patterns (Hawrylycz et al. 2015; Richiardi et al. 2015; Vértes et al. 2016; Wang et al. 2015). While these studies suggest that gene expression and aspects of brain microstructure have similar spatial patterns, it is unclear to what extent they covary within a particular brain region or across regions.

The relationships of molecular and neuroimaging features with brain disease or other phenotypes have been studied independently. For instance, identifying brain structures that covary with disease status is a common focus of neuroimaging. Likewise, identifying covariation of gene expression with disease status is a common focus of molecular biology. However, studies which span these two approaches to test the covariation of gene expression and brain structure are limited. Efforts to unite molecular biology with neuroimaging in the context of disease through “imaging genetics” have identified a small number of polymorphisms tied to variation in brain structures (Hibar et al. 2015; Munafò et al. 2008; Stein et al. 2012) including a subset of AD GWAS variants (Braskie et al. 2011; Erk et al. 2011; Kohannim et al. 2013; P. Zhang et al. 2015). However, variation in gene expression or other omics in the brain has not been measured concurrently with neuroimaging in the same set of persons. The increasing body of evidence that molecular maps of the brain correspond to structural and functional brain maps (Hawrylycz et al. 2015; Krienen et al. 2016; Richiardi et al. 2015), and the tight integration of gene expression and epigenetics with cellular function (Bishop et al. 2010; West and Greenberg 2011), indicate the potential for a unified “imaging omics” perspective on disease, using omics and imaging obtained from the same set of brains.

**Fig. 1:**
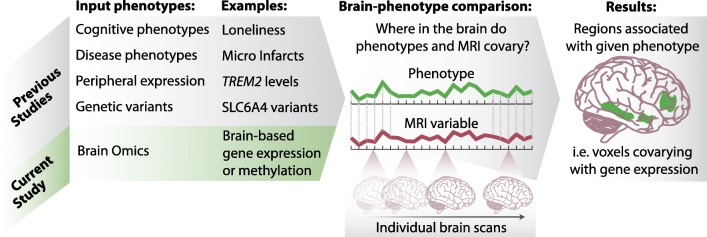
**Schematic of imaging omics, a combination of brain omics and neuroimaging, with parallel methodology to other types of imaging studies**. The current study identifies brain regions whose microstructure (as measured by MRI) are associated with brain omics data, such as gene expression or methylation, in a manner parallel to typical neuroimaging studies

We followed an imaging omics approach to discover relationships between omics and neuroimaging, using ~200 brains with paired omics and *ex-vivo* neuroimaging (Dawe et al. 2014; Dawe et al. 2009; Dawe et al. 2016; Kotrotsou et al. 2014; Kotrotsou et al. 2015), from two longitudinal cohort studies of aging (A Bennett et al. 2012; Bennett et al. 2005). Our approach to finding relationships between brain omics and structure is analogous to typical neuroimaging studies and imaging genetics studies (Fig. 1). However, instead of using genetic polymorphisms, we use gene expression and methylation data generated from the dorsolateral-prefrontal cortex (DLPFC) as our molecular trait of interest, and map it onto the brain, just as traits are mapped to the brain in a typical neuroimaging study (overview and comparison of approach in Fig. 1).

Previously we conducted expression-wide and methylation-wide association studies, testing for relationships to the transverse relaxation rate (R_2_) in cognition-associated brain regions, controlling for the effects of common age-related brain neuropathologies (Yu et al. 2017). We found four genes associated with R_2_, the inverse of T_2_. In the present study, we extend this prior work by examining molecular systems defined by DLPFC expression and methylation data in relation to a wide range of white-matter brain regions. We then use tractography to identify cortical regions connected via the white-matter regions and associated DLPFC molecular systems. Finally, we take the molecular systems that are related to brain microstructure, and examine their associations with AD clinical and pathologic traits. Based on these tests, we demonstrate the existence of several associations between molecular systems and brain microstructure. We map the spatial extent of these relationships, the genes involved and their functional characteristics, and the relevance of these imaging omics associations to common age-related neuropathologies and cognitive decline - the core clinical feature of AD.

## Methods

### Parent study and substudy characteristics

We evaluated data from two prospective cohort studies: the Religious Orders Study (ROS) (A Bennett et al. 2012), and the Rush Memory and Aging Project (MAP) (Bennett et al. 2005). ROS and MAP were designed to have consistent data acquisition and processing and are analyzed jointly in numerous publications (Arfanakis et al. 2016; Boyle et al. 2016; Buchman et al. 2012; Lim et al. 2017; Nag et al. 2015), and this extends to neuroimaging and omics assays acquired across both cohorts. ROS enrolls older religious clergy from across the United States since 1994, while MAP started in 1997 and enrolls older residents from Chicago-area retirement facilities and subsidized housing, and other older residents through church groups and social service agencies. The parent cohort studies and substudies were approved by Rush University Medical Center Institutional Review Boards. Participants provided written informed consent and all participants signed an Anatomic Gift Act for brain donation.

A subset of participants in ROS and MAP underwent postmortem structural neuroimaging. RNAseq and/or DNA methylation assays were performed on DLPFC brain tissue. Data from 222 participants were used in this study, with a mean age of 89.8, of which 153 were female. These participants exhibited a typical distribution of cognitive function for their age (mean MMSE proximate to death =19.5, median = 23). Full clinical and demographic characteristics are found in Table 1.

**Table 1:** Clinical characteristics of imaging omics cohort. Full classification criteria shown in supplementary data, and raw data available to download through www.radc.rush.edu

	w/ RNAseq	w/ DNA methylation
*n*	168	222
age at death (mean, sd)	89.74 (6.10)	89.75 (5.96)
female (n, %)	114 (67.9)	153 (68.9)
years of education (mean, sd)	15.92 (3.52)	15.77 (3.59)
MMSE, last visit (mean, sd)	20.54 (9.35)	19.50 (9.68)
Clinical dementia (n, %)	75 (44.6)	112 (50.5)
MCI (n, %)	44 (26.2)	50 (22.5)
NCI (n, %)	49 (29.2)	60 (27.0)
AD (n, %)	70 (41.7)	106 (47.7)
Global AD pathology (mean, sd)	0.69 (0.60)	0.75 (0.62)
Amyloid score (mean, sd)	4.62 (4.45)	4.82 (4.52)
Tangles score (mean, sd)	6.43 (8.45)	6.89 (8.18)
Presence of gross infarctions (n, %)	53 (31.5)	72 (32.4)
Presence of microinfarcts (n, %)	46 (27.4)	57 (25.7)
Presence of Lewy bodies (n, %)	30 (17.9)	45 (20.3)

### Neuropathology protocols

Details on clinical and neuropathological methods in ROS and MAP have been extensively published (Bennett et al. 2006b; Schneider et al. 2012; Schneider et al. 2004) and data access links are provided in supplementary methods. Due to their impact on structural brain imaging we assess levels of micro and macroscopic infarcts, β-amyloid load, paired helical filament (PHF) tau tangle density, and Lewy bodies in multiple brain regions. Neurofibrillary tangles were also quantified using Braak staging and neuritic plaque frequency, according the Consortium to Establish a Registry for Alzheimer’s Disease (CERAD). Separately, a composite measure of plaques and tangles assesses global burden of AD pathology (Bennett et al. 2004). Details of all other neuropathology measures are shown in supplementary methods.

### Cognitive function assessment and clinical diagnoses

Cognitive function in ROS and MAP participants is assessed annually along multiple dimensions with 21 cognitive tests. Seventeen tests are used to create composite scores of global cognition as well as five cognitive domains of episodic memory, semantic memory, working memory, perceptual speed and visuospatial abilities. Participants are evaluated by a clinician who used cognitive and clinical data to identify AD and other dementias. Detailed methods are published (R. Wilson et al. 2010; R. S. Wilson et al. 2015; R. S. Wilson et al. 2007) and are provided in Supplemental Methods. The clinical evaluation was done in a three stage process that involved a computer generated actuarial decision tree, followed by a clinical diagnosis by a neuropsychologist which identified the presence of cognitive impairment, followed by a clinician who identified the presence of dementia and its causes. MCI refers to those persons with cognitive impairment without dementia. Details have been previously published (Bennett et al. 2006a; Bennett et al. 2002). After death, a neurologist reviews all clinical data blind to pathologic data and makes a summary clinical diagnosis. Of the 222 participants in this substudy, 60 had no cognitive impairment, 49 had MCI, 106 had AD and 6 had other dementia.

### Generation of RNAseq and methylation data

Details on RNAseq and methylation data are published (De Jager et al. 2014; Ng et al. 2017). Briefly, RNA from 168 individuals was extracted from DLPFC with the miRNeasy mini kit (Qiagen, Venlo, Netherlands) and the RNase free DNase Set (Qiagen, Vento, Netherlands). RNA concentration was quantified using Nanodrop (Thermo Fisher Scientific, Waltham, MA), and RNA quality was assessed using an Agilent Bioanalyzer. RNAseq was performed using Illumina HiSeq with 101 bp paired-end reads with an average depth of 90 m reads. The trimmed reads were aligned to the reference genome using Bowtie and the expression FPKM values were estimated using RSEM; see supplement for normalization details.

DNA from 222 individuals was extracted from DLPFC using the Qiagen QIAamp DNA mini protocol. DNA methylation data were generated using Illumina Infinium HumanMethylation450k Bead Chip assay. Raw data were further processed using Methylation Module v1.8 from the Illumina Genome Studio software suite to generate a beta value for each cytosine guanine dinucleotide (CpG); see supplement for normalization details.

### Covariates

Age is calculated from birth date and date of death; sex and years of education were self-reported from the baseline evaluation, and their effects were removed from the R_2_ signal with a linear model.

### Omics data processing

For both gene expression and methylation, we follow the standard practice of reducing the dimensionality of gene expression and methylation, by collapsing them into a smaller number of molecular systems, identified via gene coexpression or comethylation. Gene coexpression is a standard methodology for identifying functionally related gene sets, in a manner that is strictly data-driven, and which can be related to any other phenotype recorded for the cohort, such as neuroimaging. This fundamental approach, which has now been used in numerous studies (Langfelder and Horvath 2008; B. Zhang and Horvath 2005), helps to identify more robust signals in gene expression data compared to single gene approaches, and reflects the activity of multiple regulatory mechanisms (Gaiteri et al. 2014). Coexpressed gene sets are sometimes referred to as “modules”, because they are detected as clusters in the gene-gene correlation matrix. The gene coexpression methodology has been extended to DNA methylation, identifying loci and nearby genes whose methylation level fluctuate in sync, across many subjects (Numata et al. 2012). To robustly identify coexpressed or comethylated gene sets, we use a consensus clustering method (Gaiteri et al. 2015) that operates on the gene-gene Pearson correlation matrix (or CPG-CPG correlation matrix) to find gene sets whose expression or methylation levels covary across subjects. Average levels of these 47 gene and 58 methylation sets were then related to neuroimaging in the same cohort as described below.

### Neuroimaging data processing

*Ex-vivo* MRI scans show high correlation with antemortem imaging (Dawe et al. 2016) and were conducted on a 3 Tesla MRI scanner using a 2D fast spin-echo sequence with multiple echo-times (TEs), producing estimates of relaxation rates (R_2_) for each voxel. R_2_ values are the inverse of T_2_ values. Variation in voxel R_2_ values related to the molecular environment and molecular motion within a given voxel (Brown et al. 2014) such as cellular density, myelin content, or water content. R_2_ values are responsive to changes within healthy brains (Whittall et al. 1997) or certain brain injuries (Assaf et al. 1997) or disease (Briellmann et al. 2002; Fisniku et al. 2008). All R_2_ maps were warped into the space of a cerebral hemisphere template constructed from the images of 30 representative specimens, first using linear and then nonlinear registration methods.

To obtain the lists of predicted most-affected single gray matter regions (Tables S1, S3) we use the IIT Human Brain Atlas (Varentsova et al. 2014) (www.nitrc.org/projects/iit) and the *regionstat* tool to first generate the pairs of gray matter regions most likely connected by white matter fibers traversing through the white matter region of interest, and then derive single gray matter regions with the most streamlines through the white matter region of interest. Specifically, the impact score for a single gray matter region is computed by summing the percentages of streamlines traversing through the white matter region of interest and terminating to that gray matter region. For full details of neuroimaging methods, see Supplemental methods.

### Imaging omic maps

The average values for each of the resulting gene sets/molecular system is mapped onto the MRI data of brain scans of the same individuals to identify significantly correlated voxels (Fig. 1). Specifically, we identify all voxels in the cerebral hemisphere template significantly correlated with any module, under a false discovery rate (FDR) of 5%. We further guard against false positives by accepting only clusters of 100 or more contiguous voxels (100 mm^3^) that all surpass the FDR-corrected critical *p*-value. The locations with Pearson correlations that pass these criteria define locations where brain microstructure is related to the average level of a given molecular system. This methodology is parallel to standard neuroimaging studies to identify brain regions that are synchronized with the phenotype of interest; in this case the phenotype of interest is the level of gene expression or DNA methylation in various molecular systems.

## Results

### Identification of brain regions associated with molecular systems

**Fig. 2:**
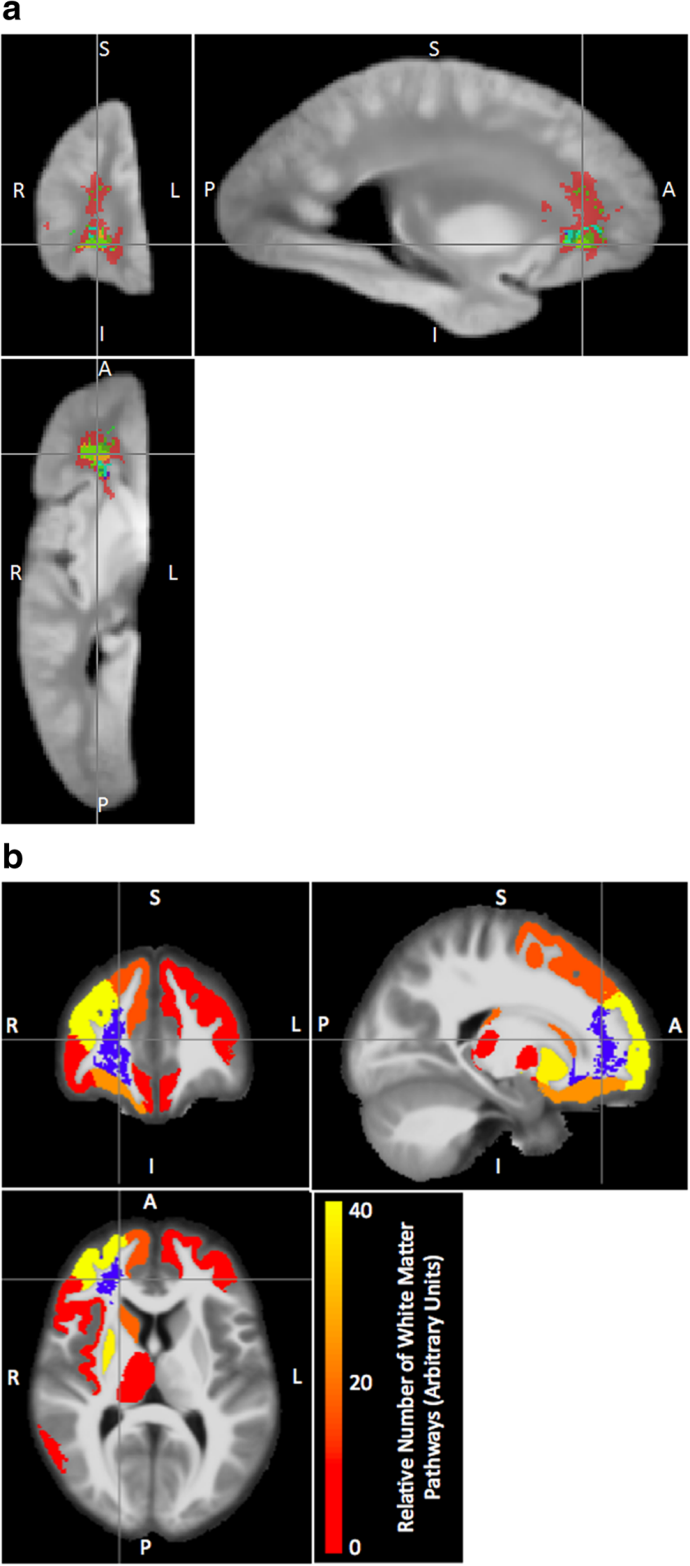
**Maps of expression-correlated brain areas ** (**a**) Locations in which *ex-vivo* transverse relaxation (R_2_) is correlated with expression of molecular systems related to transcriptional regulation (m109 - red), synaptic transmission (m23 - dark blue), myelination (m110 - green), nuclear processes (m111 - yellow) or unknown processes (m131 - light blue). All colored labels are displayed with 50% transparency to allow for partial visualization of overlapping labels. Critical *p*-value threshold for expression-correlated voxels determined by correction for multiple comparisons to yield false discovery rate of 0.05 (**b**) Cortical and subcortical gray matter terminals of white matter pathways traversing through the region with R_2_ values linked to expression of m109. The blue region comprises voxels for which R_2_ was significantly associated with the expression of m109, after FDR correction for multiple comparisons, thresholding based on a minimum cluster size of 100 mm^3^. The red-to-yellow color assigned to each gray matter region indicates the relative proportion of streamlines (a proxy of white matter fiber tracts) traversing the region in blue and terminating to that gray matter region (see also Tables S1, S2)

**Fig. 3:**
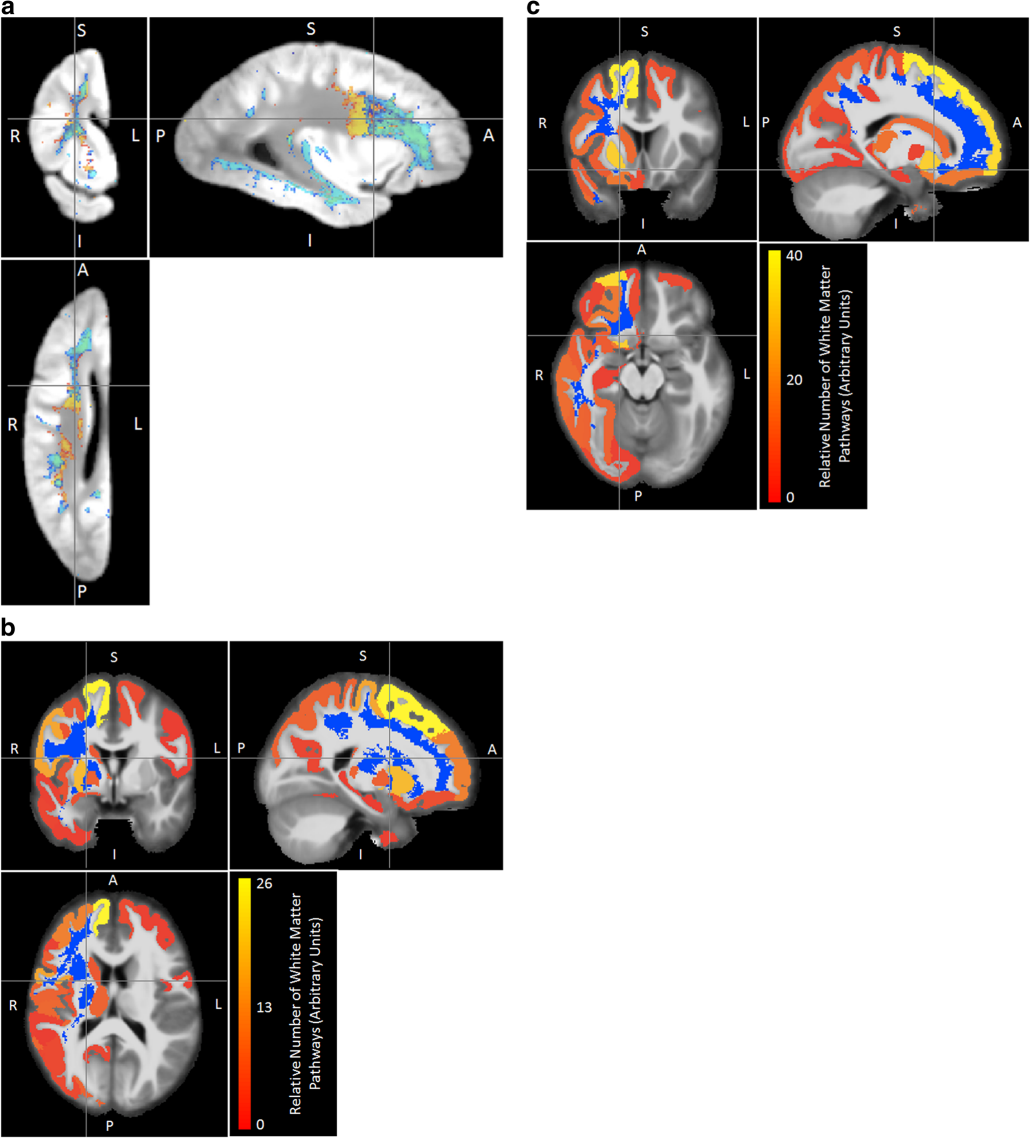
**Maps of methylation-correlated brain areas** (**a**) Locations in which *ex-vivo* transverse relaxation (R_2_) is correlated with methylation of molecular systems related to cell motility (m66 in blue-green) and a module with unknown functions (m33 in yellow). **b** Cortical and subcortical gray matter terminals of white matter pathways traversing through the region with R_2_ values linked to expression of comethylation module m33. **c** Cortical and subcortical gray matter terminals of white matter pathways traversing through the region with R_2_ values linked comethylation module m66

Analogous to imaging genetics studies, we compare the average expression of molecular systems in gene expression and DNA methylation to each neuroimaging voxel to create maps of the brain regions related to these phenotypes. Specifically, we compare coexpression or comethylation modules and brain R_2_, and observe correlations proximal to the dorsolateral prefrontal cortex, and also in voxels located in distant regions of the brain (Figs. 2a, 3a). The maps of correlations between molecular systems and R_2_ (Figs. 2a, 3a) indicate where the brain microstructure changes concurrently with the average level of a given molecular system.

**Fig. 4:**
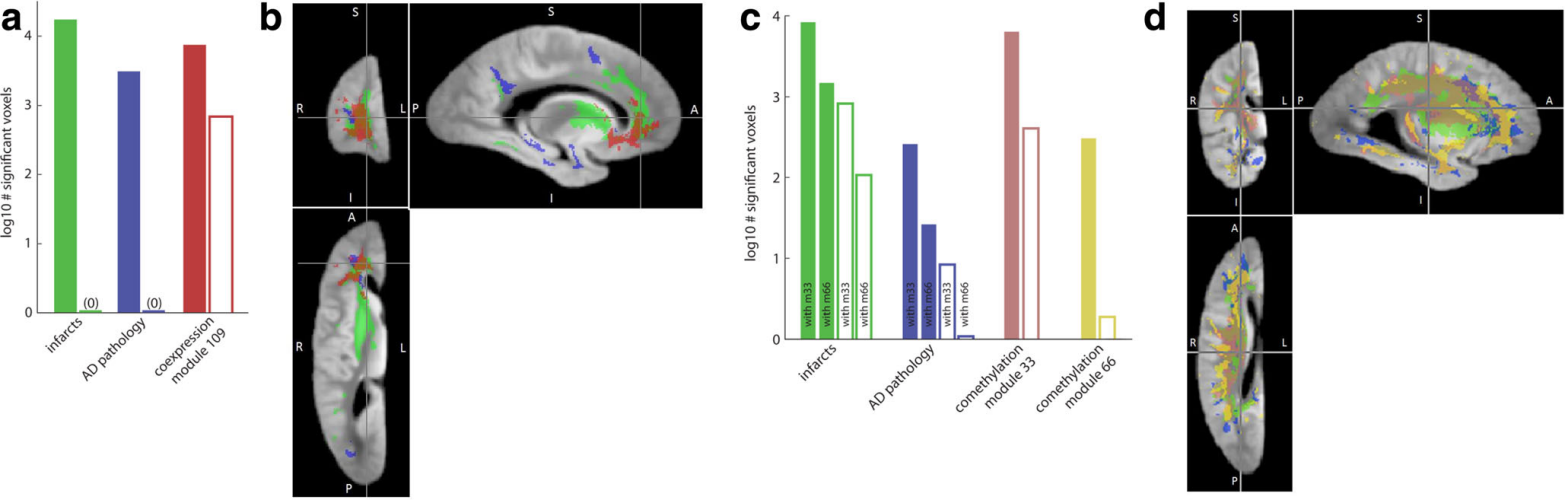
**Spatial extent and phenotypic correlates of molecular systems in expression and methylation** (**a**) Comparison of extent of voxel correlations of neuropathologies and a coexpression modules (m109) at an uncorrected *p* < .01 threshold (solid bars) and a more stringent FDR = 5% threshold (hollow bars). Neuropathology measures are controlled for m109 and m109 is controlled for neuropathologies. **b** Brain regions associated with neuropathologies and m109. Color-coding matches panel 4A, with AD pathology in dark blue, global infarcts in green, coexpression m109 in red. **c** Comparison of extent of voxel correlations of neuropathologies and comethylation modules at uncorrected p < .01 threshold (solid bars) and more stringent FDR = 5% threshold (hollow bars). In this comparison, neuropathology measures are controlled for comethylation modules and modules are controlled for neuropathologies. **d** Brain regions associated with neuropathologies and comethylation modules. Color-coding matches panel 4C, with AD pathology in dark blue, global infarcts in green, comethylation m33 in pink and comethylation m66 in yellow. The distribution and extent of pathology appears to shift between 4B and 4D due to different number of subjects, and different covariates (expression vs methylation) included in model

Because R_2_ measurements are most sensitive to changes in white-matter, we integrate atlas-based tractography to associate these changes with gray matter regions that represent the origins or destinations of the white matter fibers (Figs. 2b, 3b, c, Tables S1-S6, see methods). The brain regions likely related to levels of coexpressed molecular systems are primarily located in the frontal cortex, but regions connected via the impacted white matter also include subcortical structures, particularly the putamen (Tables S1, S2, Fig. 2b). The white matter regions associated with comethylation (Fig. 3a) are more extensive (Fig. 4) than those associated with coexpression (Fig. 2a). The gray matter regions predicted to be connected by methylation-associated white matter regions include a comprehensive range of frontal cortex regions (Fig. 3b, Tables S3-S6), as well as some temporal and parietal areas. The relatively unique predicted effects on multiple temporal regions and the precuneus stem from a specific comethyation system associated with cell morphology (Fig. 3, Tables S5, S6).

### Functional properties of molecular systems associated with neuroimaging

Although the molecular systems associated with R_2_ are enriched with a diverse set of molecular functions (Tables S9, S10), despite being derived without reference to any ontology (Tables S8, S9). For instance, the coexpression m109 module is enriched for transcriptional regulatory systems (*p* < 10^−7^, Table S9); the m66 comethylation module is enriched for ontology categories generally related to neurogenesis and morphogenesis around synapses (p < 10^−5^ Table S10). Several modules are highly enriched for specific cell types, such as microglia. However, those most highly enriched cell type modules *do not* have omic correlations in this dataset (Tables S11, S12). This implies that cell-type specificity is not sufficient to produce imaging omic associations. The lack of cell type enrichment in methylation modules indicates that cell type signatures alone are also not necessary for the existence of imaging omic associations.

**Fig. 5:**
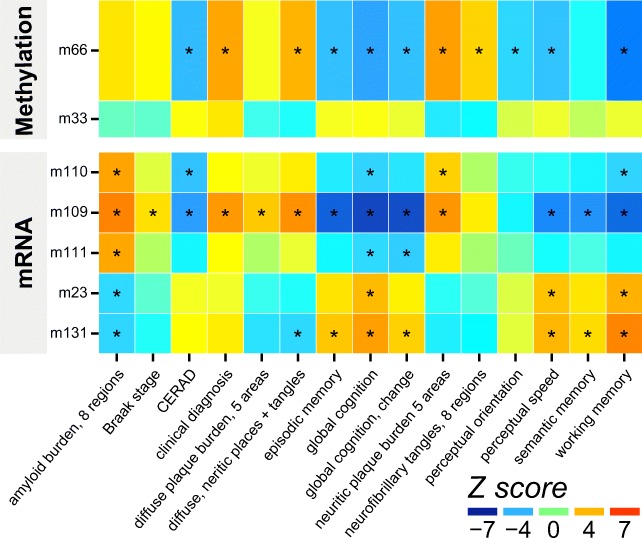
**Module-trait associations of modules with neuroimaging correlations.** Full trait descriptions in supplement. Asterisks (*) denote associations significant at FDR of 5%

In addition to their correlations with R_2_ values in extensive brain areas, both coexpression module m109 (transcriptional regulation) and comethylation module m66 (synapses and morphogenesis) have significant correlations with a wide range of AD clinical and pathologic phenotypes, including β-amyloid load and tau tangle density, and with AD diagnosis, global cognition and cognitive systems (Fig. 5). The consistency of correlations across multiple subcomponents of cognition, and various assays of AD pathology confirms the robustness of the findings. We also validated the relevance of m109 to AD pathology *in vitro* by testing the effects of influential genes within the module on Aβ42 levels in astrocyte cultures, showing significant effects for *INPPL1* and *PLXNB1* (Mostafavi et al. 2018).

### Comparison to neuroimaging of age-related pathologies

To provide context for the spatial extent and statistical strength of these imaging omic associations, we contrast them with voxels correlating with common neuropathologies (Fig. 4b, d). The number of voxels associated with select molecular systems is commensurate with the number of voxels associated with neuropathology; including those neuropathologies with the strongest relationships with imaging, such as gross infarcts and AD pathology (Fig. 4a, c). While expression-related regions are largely frontal, methylation-associated regions cover both temporal and frontal regions that are typically associated with AD pathology. Because pathological processes have an effect on both omics and brain characteristics, we check to what extent pathology may account for imaging correlations. To do this we account for the effects of all neuropathologies with known large effects on structural imaging: AD, infarcts, and Lewy body disease (Dawe et al. 2014). This reduces the number of voxels associated with m109, but relationships remain numerous, and focused on the same region (Figs. 4a, S1). By contrast, including gene expression in a model of R_2_ values weakens the associations between R_2_ and neuropathologic indices such that they do not meet the FDR 5%-corrected significance threshold (Fig. 4a). Therefore, known pathology measures do not account for a substantial proportion of the strongest imaging expression relationship. Similarly accounting for pathology reduces the number of voxels associated with comethylation module m66, but they remain significant and focused in the same region. However, the number of voxels associated with m33 is only slightly reduced when accounting for pathology (Fig. 4c), indicating it represents a non-disease or basic biological relationship between DNA methylation and brain microstructure.

Finally, because m109- and cognition-associated brain regions are semi-overlapping (Fig. S2) we compare the variance in cognitive decline explained by this single module compared to mean R_2_ values from all voxels that are correlated with cognition. We find that this single coexpression module explains 17% of the variance in cognitive decline and an additional 7% of the variance in cognitive decline above and beyond averaged R_2_ values of brain regions associated with cognition.

## Discussion

Using brain gene expression, methylation and *ex-vivo* MRI data from two longitudinal cohort studies of aging, we find molecular systems synchronized with MRI-derived molecular brain characteristics in many brain areas. These imaging-omic associations are spatially extensive and involve hundreds of genes in several molecular systems, including transcriptional regulation and cell morphology. Further, both the molecular systems and the brain regions with which they covary are associated with a wide range of AD clinical and pathologic phenotypes suggesting that both have important functional consequences.

Molecular systems and brain microstructure are independently known to be essential factors in cognitive function and disease susceptibility. The scope and strength of their coordination has been challenging to observe, although glimpses of large-scale coordination have emerged (Fulcher and Fornito 2016; Hibar et al. 2015; Krienen et al. 2016; Richiardi et al. 2015). To date, such studies have focused on comparing distributions of gene expression from one data source with neuroimaging features acquired from another data source. By obtaining omics from the brain paired with neuroimaging from a large cohort we follow the typical neuroimaging approach of identifying brain regions that covary with molecular levels. Our results expand prior work in several important ways.

First, we are able to demonstrate the existence of covariation between two omics and brain structures. The molecular systems are identified without reference to neuroimaging, pathological variables or molecular ontologies; they are produced by the activity of regulatory systems and are highly reproducible (Gaiteri et al. 2014; Mostafavi et al. 2018). The specific MRI measure of R_2_ characterizes the molecular environment and molecular motion within a given voxel (Brown et al. 2014) such as cellular density, myelin content, or water content. These R_2_ values are responsive to changes within healthy brains (Whittall et al. 1997), certain brain injuries (Assaf et al. 1997) or disease (Briellmann et al. 2002; Fisniku et al. 2008). As a result, our findings on imaging omic association identify areas of the brain in which expression or methylation of a given molecular system is associated with R_2_ values and the molecular environment they represent in a given brain area. This also indicates the interoperability of omic and neuroimaging perspectives on brain diseases, particularly within select molecular systems and brain regions.

Second, these imaging omics relationships have a practical influence on AD and likely other neuropathologies and diseases. Here they provide a coherent representation of the molecular and brain integrity progression of AD, prioritizing specific molecular systems as targets, identifying their spatial correlates in the brain, and tracking how these relate to regional correlates of neuropathology and cognition. By bringing together molecular and neuroimaging perspectives on AD it may be possible to merge their strengths as drug development tools and biomarkers for a more efficient perspective on pathogenesis. Thus neuroimaging results are no longer isolated from molecular interventions, but it will be known that structural brain changes in a given region may be controlled by molecular levels nearby or elsewhere in the brain.

Third, we explore the potential biological basis of imaging omic relationships. The causality behind brain omic imaging associations is more complex than that of imaging genetics, as feedback loops, such as activity-driven expression, are pervasive in the brain (West and Greenberg 2011). If omic systems are generally upstream, those molecular systems may be useful in controlling the molecular changes observed through MRI. If they are downstream of these brain changes, the molecular systems mark response to a process in a particular brain area. We explore the possibility that some third factor, such as pathology, may jointly influence brain omics and imaging. However, pathology has limited responsibility for some imaging omic associations and other imaging omic associations persist after controlling for several common neuropathologies. The distribution of cell types is also unlikely to completely account for our findings. Thus, imaging omic relationships appear to have relevance both to disease neurobiology and basic brain function.

The strength of these results is made possible by post-mortem neuroimaging on subjects with multiple brain omics, while their robustness is supported by the large cohort size and many detailed neuropathology and cognitive assessments. These diverse sources of information allow us to explore the basis for synchronization between brain omics and neuroimaging, which may be partially disease-driven, but also disease-independent. Limitations and open questions on the current study include the causality between brain microstructure and the molecular levels assayed by omics, and also questions of the extent of imaging omic relationships in various forms of neuroimaging. As persons in the parent cohorts are currently undergoing ante-mortem imaging, future studies can examine the fMRI associations of omics. Future *in-vitro* experiments, evaluating cell morphology before and after perturbing key genes in imaging-associated systems, will be helpful in dissecting the causality of imaging omics relationships. Omics data from additional brain regions, or additional types of neuroimaging, may potentially show similar or unique imaging omic association maps.

## Electronic supplementary material

ESM 1 (DOCX 226 kb)

ESM 2 (XLSX 271 kb)
