# Supplementary material for: Gene expression and DNA methylation are extensively coordinated with MRI-based brain microstructural characteristics
Source: Brain Imaging Behav. 2018 Jun 22;13(4):963–72. doi: 10.1007/s11682-018-9910-4 (PMC6309607; doi:10.1007/s11682-018-9910-4)
Supplement: Supplementary file 1 — (DOCX 226 kb) [file 11682_2018_9910_MOESM1_ESM.docx]

**Supplemental methods**

*Cohort summary - cognitive and neuropathological phenotypes*

We examine the relationship between gene expression and MRI R_2_ voxel values in the context of two longitudinal, community-based aging studies: the Religious Orders Study (ROS) and the Rush Memory and Aging Project (MAP). Together, these ongoing studies have enrolled ~3000 older persons without dementia all of whom have agreed to brain donation and annual detailed clinical evaluation, cognitive testing and blood donation. From the ROSMAP cohorts we acquired n=168 paired *ex-vivo* T_2_-weighted MRI scans and RNAseq-based gene expression from the dorsolateral prefrontal cortex (DLPFC), and n=222 Illumina 450K methylation assays, also from the dorsolateral prefrontal cortex, paired with *ex-vivo* T_2_-weighted MRI scans (n=159 persons supplying both transcriptome and methylome data).

*Β-Amyloid and Tau measurement*

To quantify the burden of parenchymal deposition of β-amyloid and the density of abnormally phosphorylated paired helical filament tau (PHFtau)-positive neurofibrillary tangles, tissue was dissected from eight regions of the brain: the hippocampus, entorhinal cortex, anterior cingulate cortex, midfrontal cortex, superior frontal cortex, inferior temporal cortex, angular gyrus, and calcarine cortex. 20µm sections from each region was stained with antibodies to the amyloid beta protein and the tau protein, and quantified with image analysis and stereology, as previously described^1, 2^. Briefly, β-amyloid was labeled with an antibody for beta-amyloid (10D5; Elan, Dublin, Ireland; 1:1,000). Immunohistochemistry was performed using diaminobenzidine as the reporter, with 2.5% nickel sulfate to enhance immunoreaction product contrast. Between 20 and 90 video images of stained sections were sampled and processed to determine the average percent area positive for β-amyloid. PHFtau tangles were labeled with an antibody specific for phosphorylated tau (AT8; Innogenetics, San Ramon, CA; 1:1,000). Between 120 and 700 grid interactions were sampled and processed, using the stereological mapping station, to determine the average density (per mm^2^) of PHFtau tangles. The scores across the eight regions were averaged, for β-amyloid and PHFtau separately, to create a single summary measure for each protein.

For each participant, comprehensive cognitive assessments were administered at baseline and during each annual follow-up visit. Details on cognitive assessment have been described previously^3, 4^. Briefly, the battery contains a total of 17 cognitive performance tests which assess 5 dissociable cognitive domains including, episodic memory (7 measures), semantic memory (3 measures), working memory (3 measures), perceptual speed (2 measures), and visuospatial ability (2 measures). To minimize the floor and ceiling effects, composite measures were used to examine the longitudinal cognitive decline. For each test, raw scores were standardized using the baseline mean and SD across the cohorts. The z-scores were subsequently averaged across all the 17 tests to obtain a summary measure representing global cognition. Similarly, summary measures for individual cognitive domains were obtained by averaging z scores from the corresponding tests. The longitudinal rate of decline was computed for each participant using linear mixed models, which estimate the mean rate of change for the sample as a whole, but allow positive or negative deviations for each individual and are less sensitive to the number of follow-up visits or missing data.

*RNAseq processing*

RNAseq samples for persons with paired neuroimaging were subset from a larger (n=540) cohort with biopsies of gray matter of dorsal lateral prefrontal cortex (DLPFC). These samples were extracted using Qiagen's miRNeasey mini kit (cat. no. 217004) and the RNase free DNase Set (cat. no. 79254). RNA was quantified using Nanodrop. Quality of RNA was evaluated by the Agilent Bioanalyzer. All samples were chosen to pass two initial quality filters: RNA integrity (RIN) score >5 and quantity threshold of 5 ug (and were selected from a larger set of 724 samples). RNA-Seq library preparation was performed using the strand specific dUTP method with poly-A selection. Samples were sequenced with a target coverage of 50M reads; the mean coverage for the samples passing QC is 95 million reads (median 90 million reads).

RNA-Seq data were processed by our parallelized pipeline. This pipeline includes trimming the beginning and ending bases from each read, identifying and trimming adapter sequences from reads, detecting and removing rRNA reads, and aligning reads to reference genome. The non-gapped aligner Bowtie was used to align reads to the transcriptome reference, and RSEM was used to estimate expression levels for all transcripts. The FPKM values were the outcome of our data RNA-Seq pipeline.

To remove outlier samples based on quantified expression profiles, following a previous approach, the D-statistic was computed as the median correlation of all genes (based on expression profiles) of each sample with all other samples. An additional 13 samples with D-statistics < 0.9 were deemed outliers and excluded.

We applied quantile normalization to FPKM first and then used the combat algorithm to remove potential batch effect. Expression levels were quantified for 55,889 unique genes and 190,051 transcripts. We placed a threshold for expression, only keeping genes with at least 4 reads in 100 individuals (yielding 13,484 significantly expressed genes).

After quantile normalization and batch correction, we used linear regression (on log2 expression data) to remove the effect of major biological and technical confounding factors on a per-gene basis. Biological confounding factors include three genotyping PCs (to represent ancestry), age at death, and sex. Technical confounding factors include RIN, number of ribosomal bases, number of aligned reads, study index (ROS or MAP), and Post Mortem Interval (PMI).

*Methylation processing*

The Illumina 450K platform contains a mixture of “type 1” and “type 2” probes which have distinct methylation levels that can negatively affect analysis, so we use the wateRmelon R-package to account for this mixture and process all raw 450K arrays into Beta methylation values. Systems biology analyses that rely on covariance in high dimensional data are sensitive to technical variation or other data artifacts. Therefore, we assess the impact of known and unknown technical variation by monitoring the magnitude of principal components in the data, as technical variation is removed in the normalization. An R-package containing all methylation data, intermediate stages, final output and help pages is available to download through hub.radc.edu/omics, and includes detailed rationale for our data normalization, as well as comparisons to alternative normalizations. The final model removed two measures of experimentally-assessed neuron-to-glia ratios, which had the strongest effect on methylation of any variable, as well as variables related to the position of arrays, lab processing batch, PMI, sex and age at death.

After normalization we perform an initial data reduction using the minfi R package to collapse adjacent probes with similar methylation levels into single units. This reduces the ~450K methylation probes to 125878 CPG blocks, which we refer to simply as CPG’s. We then compute all pairwise CPG-CPG Pearson correlation values. In preparation for clustering this correlation matrix to find comethylation modules, for computational feasibility, we retain the top 1% or ~1200 most extreme CPG-CPG (weighted) correlations per CPG, setting all others to zero. Clustering this sparse, signed, weighted matrix using SpeakEasy produces 58 modules (mutually correlated CPG sets) containing at least 100 CPG’s, each associated with a proximal gene.

*Identifying molecular systems*

Comparisons between every gene (~13000 expressed genes) and methylation loci (~450,000) with every voxel (400,000) face a very large multiple testing burden. Therefore, we follow standard practice of reducing gene expression and methylation to coexpressed or comethylated systems^5^, each of which is composed of genes with similar patterns of expression or methylation, measured across all persons.

The biological origin of patterns of synchronized gene coexpression is are several cellular and molecular mechanisms such as chromosome conformation, epigenetics, and microRNA’s^6^. Similarly gene comethylation is likely generated by the action of transmethylases as well as heredity. Therefore, identifying groups of coexpressed or comethylated genes (often termed “modules”) provides a limited number of signals that are robust measures of the activity of biological processes the genes they target.

In order to statistically identify groups of coexpressed or comethylated genes we use a consensus clustering approach “SpeakEasy”^7^. This method was used because it 1) does not require any manual parameter tuning or cutoffs, as do hierarchical clustering approaches, 2) provides the highest recorded performance on synthetic clustering benchmarks, and 3) accurate recovery of biological gold standards^7^ and 4) provides clusters less likely to be influence by statistical or data artifacts due to its stochastic nature and consensus clustering. This method operates on the gene-gene or CPG-CPG Pearson correlation matrix to identify clusters/modules of coexpresssed/comethylated genes.

Consensus clustering identifies 47 coexpressed gene sets, or modules, in this dataset, many of which are enriched for particular cellular functions (Table S7). Consensus clustering operating on Illumina 450K methylation data identified 58 comethylation modules (Table S8).

The coexpression and comethyation modules are derived in isolation from any pathway or phenotypic information, however they are frequently enriched in particular molecular functions. The gene ontology categories associated with the coexpression and comethylation modules with significant neuroimaging correlations are shown in Tables S9, S10. Functional enrichment scores for gene ontology categories for each module were calculated using the R GOtools package.

*Cell type composition and relationship with gene and methylation modules*

Random variation in the proportion of cell types across samples (individuals) can lead to identification of modules that are enriched in genes that are highly expressed in particular cell types. To assess which of the modules have some relationship to cell types, we calculate the median rank of all genes in a module in the rank-ordered expression of genes in a given cell type (see below for sources of cell-type specific gene expression). We repeat this calculation for all combinations of modules and cell types. This provides an indicator of when modules contain genes that are highly expressed in a canonical cell type. Because modules generally consist of hundreds of genes, this test is well powered to detect even minor enrichment, with nearly every module showing significant enrichment for some cell type, down to the minimum p-value (based on 10,000 permutations). Therefore, we report a more practical measure of enrichment effect size, which is the median rank of genes in a module in the sorted expression of genes from a given cell type, divided by the median rank from permuted gene sets. We also report the specificity of this effect size (actual effect size/median of effect sizes for all cell types).

The basis for the cell type signatures is gene expression from mouse brain, wherein cell types are better annotated with marker genes than in humans. Specifically we utilize NeuroExpresso^8^, which is a QC’d and normalized collection of all Affymetrix 430 chip assays of nominally cell type specific experiments, which were applied to measure gene expression in a range (n=31) of cell types in the mouse brain, where expression for a given cell type is quantified by mean expression of each gene across all arrays of that cell type. These cell types were captured by a range of methods, including fluorescence assisted cell sorting and laser capture microdissection. These data may also have been generated from disease models or exposed to various perturbations.

Single-cell RNAseq is an excellent mechanism to identify cell types in an unbiased manner, but for our purpose the largest brain cell type studies in GEO do not sequence deeply enough to be useful: they show distinct expression for a few thousand genes at most, compared to the twenty thousand surveyed on the microarrays, so while they are sufficient for clustering-based cell type definitions that utilize all gene simultaneously, they are generally not sufficient for our cell typing purposes, which require varying levels of expression from a majority of the few hundred genes in a given module.

*Module-trait correlations:*

Traits are described in order listed in Figure 4, and hyperlinked to longer descriptions, found on the Rush Alzheimer’s Disease Center [Resource Hub](https://www.radc.rush.edu/), which has full details of specific tests and response processing. Unless noted, all traits denote measurements taken at last visit prior to autopsy.

[Amyloid burden](https://www.radc.rush.edu/docs/var/detail.htm?category=Pathology&subcategory=Beta-Amyloid&variable=amyloid): immunohistochemistry of amyloid average over 8 brain regions

[Braak stage](https://www.radc.rush.edu/docs/var/detail.htm?category=Pathology&subcategory=Alzheimer%27s%20disease&variable=braaksc): a 6-stage semiquantitative measure of severity of neurofibrillary tangle pathology

[CERAD](https://www.radc.rush.edu/docs/var/detail.htm?category=Pathology&subcategory=Alzheimer%27s%20disease&variable=ceradsc): semiquantitative estimates of neuritic plaque density as recommended by the Consortium to Establish a Registry for Alzheimer’s Disease (CERAD)

[Clinical diagnosis](https://www.radc.rush.edu/docs/var/detail.htm?category=Clinical%20Diagnosis&subcategory=Final%20consensus%20diagnosis&variable=cogdx): comprehensive clinical diagnosis (blind to pathology) at time of death

[Diffuse plaques](https://www.radc.rush.edu/docs/var/detail.htm?category=Pathology&subcategory=Beta-Amyloid&variable=plaq_d): average of diffuse plaques in 5 brain regions

[Diffuse, neuritic plaques and tangles](https://www.radc.rush.edu/docs/var/detail.htm?category=Pathology&subcategory=Alzheimer%27s%20disease&variable=gpath): combined measure of core AD neuropathology measures across multiple brain regions

[Episodic memory](https://www.radc.rush.edu/docs/var/detail.htm?category=Cognition&subcategory=Domains&variable=cogn_ep): composite of 7 tests for episodic memory

[Global cognition](https://www.radc.rush.edu/docs/var/detail.htm?category=Cognition&subcategory=Global%20cognition&variable=cogn_global): composite of 19 tests, with overlap with episodic, semantic and working memory variables and perceptual speed and orientation variables

[Global cognition, change](http://www.radc.rush.edu/docs/var/detail.htm;jsessionid=9F8A84F49FA17B1D609C697241633412?category=Cognition&subcategory=Estimated+slopes&variable=cogng_random_slope): slope of cognition over time (generally cognitive decline) controlling for age, sex and education

Global cognition, adjusted: slope of cognitive decline, controlling for age, sex, education and a comprehensive list of AD and AD-associated neuropathologies, including infarctions, neurofibrilary tangles, diffuse and neuritic plaques, Lewy bodies, hippocampal sclerosis, TDP43 state, cerebral amyloid angiopathy, cerebral atherosclerosis and arteriosclerosis.

[Gross chronic infarcts](https://www.radc.rush.edu/docs/var/detail.htm?category=Pathology&subcategory=Vascular%20-%20Infarcts%20(Presence%20of)&variable=ci_num2_gct): binary variable for presence of one or more gross infarcts

[Lewy body disease](https://www.radc.rush.edu/docs/var/detail.htm?category=Pathology&subcategory=Lewy%20body/PD&variable=dlbdx): 4 stages of distribution of α-synuclein in the brain

[Neuritic plaques](https://www.radc.rush.edu/docs/var/detail.htm?category=Pathology&subcategory=Beta-Amyloid&variable=plaq_n): average of neuritic plaques in 5 brain regions

[Neurofibrilary tangles](https://www.radc.rush.edu/docs/var/detail.htm?category=Pathology&subcategory=PHF%20tau%20tangles&variable=tangles): average of neurofibriliary tangles across 8 brain regions

[Perceptual speed](https://www.radc.rush.edu/docs/var/detail.htm?category=Cognition&subcategory=Domains&variable=cogn_ps): composite of 4 tests for perceptual speed

[Perceptual orientation](https://www.radc.rush.edu/docs/var/detail.htm?category=Cognition&subcategory=Domains&variable=cogn_po): composite of 2 visuospatial tests

[Semantic memory](https://www.radc.rush.edu/docs/var/detail.htm?category=Cognition&subcategory=Domains&variable=cogn_se): composite of 3 semantic memory tests

[Working memory](https://www.radc.rush.edu/docs/var/detail.htm?category=Cognition&subcategory=Domains&variable=cogn_wo): composite of 3 tests for working memory

*Brain imaging*

The neuroimaging features, with which modules are compared, originate from *ex-vivo* MRI of the same brains, but contralateral hemisphere in which gene expression the RNAseq is measured. Although brain tissue undergoes considerable changes after death, previous studies indicate that *ex-vivo* volumetric^9^ and relaxometric (R_2_)^10^ measures remain well correlated with analogous antemortem measures. Therefore, like postmortem gene expression, *ex-vivo* MRI potentially offers an opportunity to assess the antemortem brain state^11^. R_2_ values summarizing the transverse relaxation rate are estimated for approximately 400,000 voxels in the cerebral hemisphere template based on spin echo images acquired at different echo times. R_2_ values are largely driven by the molecular environment and molecular motion, such as interaction of water protons with biomolecules and therefore in some cases may represent cellular density, myelin content, and water content of each voxel. These voxel-wise R_2_ values can be correlated with the average expression of each module, resulting in brain-wide maps of expression-structure relationships.

*Post-mortem imaging methods*

After a participant’s death, an autopsy technician removed the brain. One cerebral hemisphere was immersed in phosphate-buffered 4% paraformaldehyde solution and refrigerated at 4 °C within 30 min after removal from the skull. Prior to *ex vivo* MRI at approximately 30 days postmortem, the hemisphere was positioned in a container filled with 4% paraformaldehyde solution with its medial aspect facing the bottom of the container, and returned to room temperature. *Ex vivo* MRI scans were conducted on a 3 Tesla MRI scanner using a 2D fast spin-echo sequence with multiple echo-times (TEs). The relaxation rate R_2_ was measured voxel-wise from the data on multiple TEs. To facilitate voxelwise analyses, all R_2_ maps were warped into the space of a cerebral hemisphere template constructed from the images of 30 representative specimens, first using linear and then nonlinear registration methods. Any effects of age, sex and education were removed from R_2_ values with a linear model.

To obtain the lists of predicted most-affected single gray matter regions (Tables S1,S3) we use the IIT Human Brain Atlas^12^ ([www.nitrc.org/projects/iit](http://www.nitrc.org/projects/iit)) and *regionconnect* tool to first generate the pairs of gray matter regions most likely connected by white matter fibers traversing through the white matter region of interest, and then derive single gray matter regions with the most streamlines through the white matter region of interest i.e., Table S1 is derived from the full data used to generate Table S2. Specifically, the “impact score” for a single gray matter region is computed by summing the percentages of streamlines traversing through the white matter region of interest and terminating to that gray matter region.

*Imaging omic associations*

To identify neuroimaging features associated with modules, we conduct voxel-wise analyses throughout the entire cerebral hemisphere template, followed by correction for multiple comparisons via acceptance of a false discovery rate of 5%. We further guard against false positives by accepting only clusters of 100 or more contiguous voxels (100 mm^3^) that all surpass the FDR-corrected critical p-value. This allows us to identify specific cellular processes that are associated with brain structure in distant regions, such as module ‘x’ with brain voxel ‘y’.

*Comparison of effects of RNAseq vs cognition on brain imaging*

Cognitive decline has a stronger association with gene expression in human brains compared to amyloid or tau tangles (Figure 4) and also has strong association with *ex-vivo* MRI measures^10^. Therefore we compare the associations of imaging and gene expression on cognition. In this comparison we focus on m109, because it shows the highest correlation with cognition of any module (in review, Nature Neuroscience) and the majority of m109-associated voxels are also associated with cognitive decline (Figure S2). In the area of spatial overlap between m109- and cognition-associated regions, gene expression or imaging explain a similar portion of the variance in cognitive decline (16 vs. 17%, respectively). However, they explain largely different components of cognition, as their total explained variance is markedly greater (23%) than what would be expected if these factors were entirely duplicative of each other.


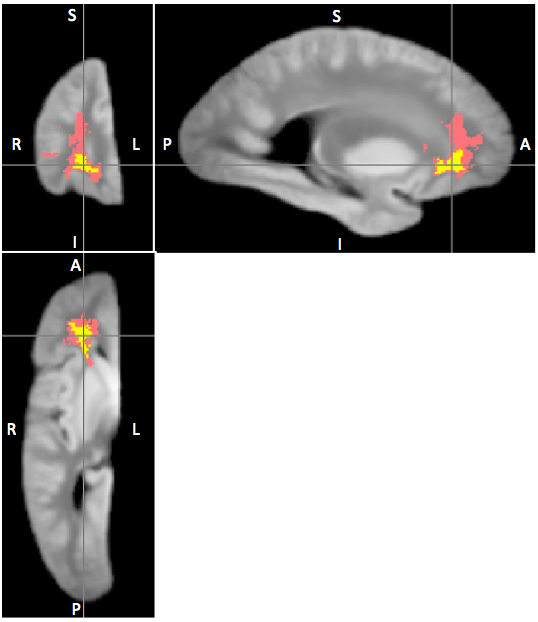


**Figure S1. Comparison of voxels correlated with m109 before and after adjusting for neuropathology.** After FDR correction for multiple comparisons, m109 expression but not AD or cerebrovascular pathology retained significant association with R_2_. The region in which R_2_ was associated with m109 expression before (salmon) and after (yellow) controlling for AD, CVD, and Lewy body pathology.


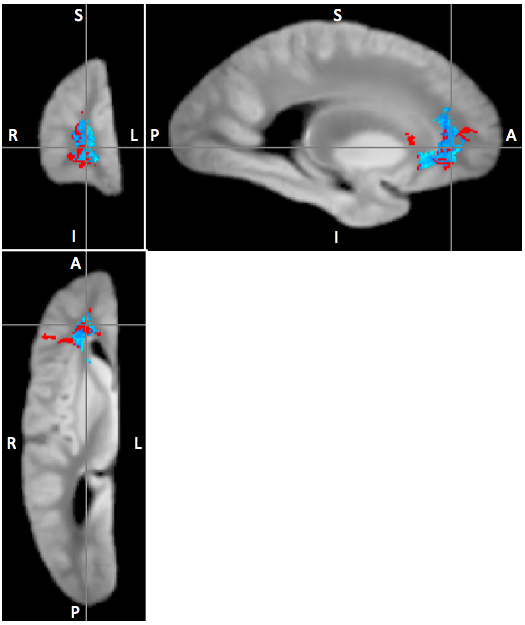


**Figure S2. Association of R_2_ with cognitive decline.** Within the region in which R_2_ is associated with expression of m109 (red), there exist voxels for which R_2_ is also associated with cognitive decline (blue). In this case, FDR correction for multiple comparisons of the R_2_-cognition association was based only on the voxels within the red region, leading to a much less stringent correction than that based on all voxels throughout the brain.

Table S1. Cortical and subcortical gray matter regions with connectivity predicted affected by expression-associated R_2_ based on combining tractography and voxels correlated with coexpression module m109.

Table S2. Pairs of cortical and subcortical gray matter regions with connectivity predicted affected by expression-associated R_2_ based on combining tractography and voxels correlated with coexpression module m109.

Table S3. Cortical and subcortical gray matter regions with connectivity predicted affected by expression-associated R_2_ based on combining tractography and voxels correlated with comethylation module m33.

Table S4. Pairs of cortical and subcortical gray matter regions with connectivity predicted affected by expression-associated R_2_ based on combining tractography and voxels correlated with comethylation module m33.

Table S5. Cortical and subcortical gray matter regions with connectivity predicted affected by expression-associated R_2_ based on combining tractography and voxels correlated with comethylation module m66.

Table S6. Pairs of cortical and subcortical gray matter regions with connectivity predicted affected by expression-associated R_2_ based on combining tractography and voxels correlated with comethylation module m66.

Table S7. Identity of genes in coexpression modules with significant imaging omic relationships.

Table S8. Identity of genes in loci of comethyation modules with significant imaging omic relationships.

Table S9. Gene ontology functions for coexpression modules with significant imaging omic relationships.

Table S10. Gene ontology functions for comethyation modules with significant imaging omic relationships.

Table S11. Cell type overlap of imaging-associated coexpression modules (m23, m109, m110, m111, m131) - results for all modules shown for context

Table S12. Cell type overlap of imaging-associated comethylation modules (m33, m66) - results for all modules shown for context

1. Boyle PA, Wilson RS, Yu L, Barr AM, Honer WG, Schneider JA *et al.* Much of late life cognitive decline is not due to common neurodegenerative pathologies. *Annals of neurology* 2013; **74**(3)**:** 478-489.

2. Schneider J, Arvanitakis Z, Yu L, Boyle P, Leurgans S, Bennett D. Cognitive impairment, decline and fluctuations in older community-dwelling subjects with Lewy bodies. *Brain* 2012; **135**(10)**:** 3005-3014.

3. Wilson RS, Beckett LA, Barnes LL, Schneider JA, Bach J, Evans DA *et al.* Individual differences in rates of change in cognitive abilities of older persons. *Psychology and aging* 2002; **17**(2)**:** 179.

4. Wilson RS, Barnes LL, Krueger KR, Hoganson G, Bienias JL, Bennett DA. Early and late life cognitive activity and cognitive systems in old age. *Journal of the International Neuropsychological Society* 2005; **11**(04)**:** 400-407.

5. Zhang B, Horvath S. A general framework for weighted gene co-expression network analysis. *Statistical applications in genetics and molecular biology* 2005; **4**(1)**:** 1128.

6. Gaiteri C, Ding Y, French B, Tseng GC, Sibille E. Beyond modules and hubs: the potential of gene coexpression networks for investigating molecular mechanisms of complex brain disorders. *Genes, Brain and Behavior* 2014; **13**(1)**:** 13-24.

7. Gaiteri C, Chen M, Szymanski B, Kuzmin K, Xie J, Lee C *et al.* Identifying robust communities and multi-community nodes by combining top-down and bottom-up approaches to clustering. *Scientific reports* 2015; **5**.

8. Mancarci BO, Toker L, Tripathy S, Li B, Rocco B, Sibille E *et al.* NeuroExpresso: A cross-laboratory database of brain cell-type expression profiles with applications to marker gene identification and bulk brain tissue transcriptome interpretation. *bioRxiv* 2016.

9. Kotrotsou A, Schneider JA, Bennett DA, Leurgans SE, Dawe RJ, Boyle PA *et al.* Neuropathologic correlates of regional brain volumes in a community cohort of older adults. *Neurobiology of aging* 2015; **36**(10)**:** 2798-2805.

10. Dawe RJ, Bennett DA, Schneider JA, Leurgans SE, Kotrotsou A, Boyle PA *et al.* Ex vivo T 2 relaxation: associations with age-related neuropathology and cognition. *Neurobiology of aging* 2014; **35**(7)**:** 1549-1561.

11. Kotrotsou A, Bennett DA, Schneider JA, Dawe RJ, Golak T, Leurgans SE *et al.* Ex vivo MR volumetry of human brain hemispheres. *Magnetic resonance in medicine* 2014; **71**(1)**:** 364-374.

12. Varentsova A, Zhang S, Arfanakis K. Development of a high angular resolution diffusion imaging human brain template. *NeuroImage* 2014; **91:** 177-186.
